# COVID‐19 vaccination success in Bangladesh: Key strategies were prompt response, early drives for vaccines, and effective awareness campaigns

**DOI:** 10.1002/hsr2.1281

**Published:** 2023-05-17

**Authors:** Nazmunnahar, Bulbul Ahamed, Md. Anamul Haque, Md. Tanbir, A. S. M. Roknuzzaman, Rapty Sarker, Md. Rabiul Islam

**Affiliations:** ^1^ Department of Sociology, Eden Women's College National University Bangladesh Gazipur Bangladesh; ^2^ Department of Pharmacy University of Asia Pacific Dhaka Bangladesh

**Keywords:** awareness, Bangladesh, COVID‐19, COVID‐19 vaccines, immunization programs, vaccination

## Abstract

Bangladesh is located in Southeast Asia that has a high population density. It is a lower‐middle‐income country. The COVID‐19 pandemic severely impacted the nation that slowed its economic growth. It halted major industries, crippling the nation's economy. The students were uncertain after the declaration of school closures. Hospitals could not treat other patients properly due to the vast health burden of COVID‐19. Bangladesh put up a solid fight during COVID‐19 as a lower‐middle‐income country. Prompt action, early vaccination drives, effective awareness campaigns, and widespread public involvement have enabled Bangladesh to bring more than 90% of its population into COVID‐19 vaccination coverage. It was possible by the effective diplomatic and local health strategy implemented by the Bangladeshi government, the country's extensive prior experience, and its long history of achieving a high success rate in other vaccination campaigns. Bangladesh was able to flatten the curve sooner than other developed countries. Therefore, the cogs of everyday social life and the economy begin to turn once more. The strategy Bangladesh used to combat the COVID‐19 pandemic through vaccination and diplomatic policy by calling on its prior experience has the potential to serve as a model for other low‐ and middle‐income countries and an example for developed nations.

## 1 BACKGROUND

Bangladesh is a South Asian lower‐middle‐income country. It has an area of 147,570 square kilometers with over 165 million inhabitants.^1^ Bangladesh's gross domestic product (GDP) in 2021 was $416.26 billion.^2^ The country has made great strides in terms of medical facilities and services it offers. Before the development of expanding international health initiatives, the Bangladeshi government prioritized vaccines as the principal method for reducing child mortality. Hence, the current child death rate in Bangladesh is 24 per 1000 people.^3^ With the aid of the World Health Organization (WHO), Bangladesh has effectively established a surveillance system for vaccine‐preventable diseases. This contribution might boost immunization against the novel coronavirus disease 2019 (COVID‐19) in Bangladesh.^4^

The Wuhan City Health Committee discovered the first known case of novel coronavirus infection in December 2019. As of March 4, 2023, the WHO had recorded over 758 million confirmed cases of COVID‐19 and over 6.8 million related deaths.^5^ Five cases of COVID‐19 were identified in Bangladesh on March 8, 2020; 10 days later, the government reported the first death related to the COVID‐19.^6^ As of March 4, 2023, there were 2,038,229 confirmed cases in Bangladesh, resulting in the deaths of 29,445 individuals.^7^

## 2 IMPACT OF COVID‐19 IN BANGLADESH

Bangladesh has suffered extreme devastation as a result of the COVID‐19 pandemic. The country faced several challenges regarding its society, school system, and social life, all needing improvement.^8^, ^9^, ^10^, ^11^, ^12^ COVID‐19 has a considerable influence on patients' overall health, including their emotional and physical well‐being. Hospitals and other medical facilities in Bangladesh have experienced significant difficulties in treating patients during the early stage of the COVID‐19 pandemic. Hospitals suffered from shortages of ventilator support to treat the severe acute respiratory syndrome due to COVID‐19. In addition, other non‐COVID‐19 patients faced several difficulties in getting the appropriate healthcare service during the early stage of the pandemic.^13^ A large portion of Bangladesh's general population was psychologically affected due to the impact of the COVID‐19 pandemic.^14^, ^15^, ^16^ During the lockdown, approximately three out of every four people in Bangladesh were experiencing various degrees of the adverse effects of loneliness.^17^ Several previous studies reported that many people have shown symptoms of psychological distress, including posttraumatic stress disorder, anxiety, and hopelessness.^18^ Nonetheless, concerning signs indicate greater suicidal thoughts and deeds, especially in healthcare.^19^, ^20^

Bangladeshi government implemented lockdowns in a wide variety of industries during the early stage of the COVID‐19 pandemic which negatively affected the country's economy. As a result, there has been a significant drop in the country's overall productivity. Agriculture, industry, and services are Bangladesh's three most important economic sectors contributing 18%, 29%, and 53% of the country's GDP. Around 20 million people have lost their jobs due to the pandemic effect. Many self‐employed or day laborers were also temporarily unemployed.^21^ Despite this, a recent study revealed that low‐income people in Bangladesh had been subjected to significant economic repercussions as a direct result of the COVID‐19 pandemic. More specifically, the per capita income of those living in low‐income areas fell by 82% to $0.32 (United States) in early April, down from $1.30 in February when they had the highest income. In comparison, the per capita income of people living in rural poverty fell by 79% during this period.^22^ As a direct consequence of the lockdown, there has been a halt in the production of essential quantities of food and a disturbance in their availability. Therefore, food costs have increased, making it difficult for people without privilege to keep up with their previous standard of living. This is the case despite the government attempting to assist these people and find a solution to the problem by providing financial assistance.^23^, ^24^

Following the appearance of multiple worrisome incidents in Bangladesh, the government decided to suspend all educational institutions throughout the country.^25^ Everybody admitted that, at first, they were caught off guard and astonished by the sudden announcement of the closure. However, they eventually came to comprehend the critical nature of choice in light of the COVID‐19 outbreak. All seminar rooms, residential halls, and libraries promptly shuttered their doors due to a widespread panic created by COVID‐19.^19^, ^26^ Students living in university residence halls were given the very short notice and told to leave the campuses immediately and return to their hometowns or villages. So, they could only bring some of the essential items with them. The universities in question immediately halted all of their official and academic endeavors. The students were displeased, uncertain, and anxious about their studies, classes, examinations, and future careers. In addition, they were tensed and concerned about the session jam. It was discovered that there were multiple disturbances to the student's learning and academic life. They could not maintain their drive to study at home since there was no requirement for them to follow a set pattern, such as getting up and going to school.^27^, ^28^ All these have a tremendous negative effect on their mental health, future learning process, and motivation for proper study continuation.

The COVID‐19 pandemic lockdown humiliates, frightens, and terrorizes Bangladesh. The shutdown affected the activities of many hospitals and private clinics.^13^ Doctors, nurses, caregivers, police, military, bankers, and political officials were infected, isolated, and even died. Infection fears closed many suburban and rural private hospitals and clinics.^11^ Coronavirus caregivers were stigmatized in many ways. Some local graveyards denied burial of deceased persons due to COVID‐19.^29^ Customers struggle with income plummets as the government limits hawkers and roadside merchants. Several construction workers are unemployed. COVID‐19 has devastated household income and spending. Low‐income residents suffer the most.^30^ Most urbanites lost jobs and returned home, spreading illnesses. Many of them had to sell their furnishings and borrow from nongovernmental organizations (NGOs) to pay household expenditures, which harms society.^31^ Almost 10 million impoverished urban Bangladeshis faced food insecurity and malnutrition during the early stage of the COVID‐19 pandemic. Urban poor faced food insecurity, insufficiency, and malnutrition. The FAO Rapid Assessment of Food and Nutrition Security in the Context of COVID‐19 in Bangladesh indicated that urban consumer basket costs increased.^32^

## 3 COUNTERMEASURES TO ADDRESS UNFORESEEN CHALLENGES DUE TO THE COVID‐19 PANDEMIC

The government and various NGOs have implemented countermeasures to address unforeseen challenges in response to the COVID‐19 pandemic. For example, on March 17, 2020, the government of Bangladesh ordered the closure of all educational institutions. Therefore, Bangladesh's academic institutions had been shut down for an extended period.^33^ Because of the constitutional crisis, several school, college, and university administrations had chosen to launch online education systems to get their students back into the classroom during COVID‐19.^34^ In addition, the government shut down factories producing clothing and other goods to prevent overcrowding and preserve social distance among the employees. About 10 million people lost their jobs as a direct consequence of the factory closures. Furthermore, the government had declared 11.90 billion USD worth of aid packages in response to the nation's widespread socioeconomic difficulties.^35^, ^36^

Inadequate healthcare services were one of the most significant obstacles to combating COVID‐19. At the early stage, most individuals lacked knowledge about the COVID‐19 virus and were unfamiliar with proper hygiene practices and etiquette.^37^ On the other hand, several hospitals and other healthcare organizations improved the quality of COVID‐19 testing and modernized their facilities. In Bangladesh, BRAC, one of the world's largest NGOs, has carried out initiatives to increase community knowledge of COVID‐19 to take preventative and protective measures. Along with the government, various charities and social welfare groups assisted in detecting COVID‐19 patients and provided financial assistance to those patients so they could remain in quarantine for 14 days. In addition, patients infected with coronavirus were also allowed to use telemedicine services provided by local and national organizations.^38^

On February 7, 2021, Bangladesh launched a nationwide immunization campaign against COVID‐19, with the primary targets being front‐line combatants and anyone aged 40 years or older.^25^ On August 10, 2021, the Bangladesh government announced that those 18 or older could receive the vaccines for COVID‐19. Also, the government intended to bring around 10 million individuals every week into the scope of the mass immunization campaign. These programs contributed to vaccinating the vast majority of this country's population and prompted educational institutions to resume normal operations.^39^, ^40^

## 4 COVID‐19 VACCINATION JOURNEY IN BANGLADESH

Vaccination is considered as the only approach to prevent the spread of this disease.^41^ In light of this, the Bangladeshi government played an essential role in vaccinating everyone.^42^ The WHO committed to vaccinating around 40% of Bangladesh's population. The Serum Institute of India sold 700,000 doses of the Oxford–AstraZeneca vaccine to Bangladesh. In addition, the Indian government arranged for the donation of 3,300,000 doses of the AstraZeneca vaccine and the distribution of 3,329,387 doses through the COVAX program.^43^

In addition, Japan and Bulgaria donated 30,57,780 and 270,000 doses of the AstraZeneca vaccine to Bangladesh. Out of 60 million doses from China, Bangladesh purchased 29,396,350 doses, received 2,100,000 as a gift, and utilized the COVAX program to acquire another 3,471,600 doses.^44^ In addition, 49,429,940 doses of vaccines from AstraZeneca, Moderna, Pfizer, and Sinopharm had been shipped to Bangladesh through the COVAX facility.^45^ Also, 3.6 million doses of the Pfizer–BioNTech vaccine had been provided to Bangladesh as part of the COVAX initiative.^46^ Consequently, more than 160 million vaccine doses had been delivered by January 2022.^42^ As soon as COVID‐19 doses became available, the government launched a mass vaccination drive to vaccinate millions over several days, with the assistance of WHO, United Nations Children's Fund (UNICEF), and other organizations. This campaign was conducted in February 2022, when 17 million individuals were vaccinated. Throughout the first few months of the year, the immunization rate in Bangladesh grew considerably due to these activities. The second dose was available in March 2021 during this campaign.^43^

Consequently, Dhaka, the capital of Bangladesh, has received the most COVID‐19 vaccine doses since the vaccine doses reached there via COVAX.^44^ In Bangladesh, 150,049,129 individuals have taken the first dose, 131,182,263 individuals have taken the second dose, 65,672,743 individuals have taken the third dose, and 569,825 individuals have taken the fourth dose.^45^ More than 90% of the population has gotten at least one dose of the COVID‐19 vaccine in Bangladesh.^45^

## 5 FACTORS BEHIND THE HIGH COVID‐19 VACCINATION RATE IN BANGLADESH

Initially, Bangladesh intended to vaccinate 138.2 million individuals, equivalent to 80% of the population.^46^ The mass immunization campaign was kicked off on February 7, 2021, with 7 million vaccine doses developed by Oxford and AstraZeneca.^47^ In the meanwhile, the government of Bangladesh has given the go‐ahead for emergency COVID‐19 vaccinations from Sinovac, Sinopharm, Pfizer‐bioNTech, Sputnik V, and Johnson and Johnson (single dosage).^48^ There are now more than 1060 national mass immunization centers that have been built.^47^ To procure and make available a COVID‐19 vaccine from the Serum Institute of India, Bangladesh entered into a contract with a domestic pharmaceutical firm. In addition, the government developed the “Surokkha App” to register COVID‐19 vaccine recipients.^45^ To encourage more people to get vaccinated against COVID‐19, the age requirement was decreased to 18, and the vaccine was made available for free by the government.^49^

Earlier launches of vaccines have resulted in the formation of solid alliances surrounding the enlarged program on immunization (EPI). Since the beginning of its work in Bangladesh, EPI has relied on the expertise of both WHO and UNICEF. The Bangladeshi government's efforts to eradicate the disease through mass vaccination have been assisted by partners as well as personnel that has been trained and are dedicated.^50^, ^51^ The EPI of Bangladesh have been successful in achieving high immunization rates since 1974; these rates have increased from 2% in 1984 to 84% in 2019.^52^ Because Bangladesh has a long history of success to achieve high vaccination coverage against diseases that can be prevented by vaccination. The people of Bangladesh vaccinate their children using EPI and non‐EPI vaccination programs.^51^

The government of Bangladesh has begun a campaign to promote awareness of this potentially lethal disease through the media.^53^ In Bangladesh, the government and NGOs have hosted a large number of seminars, webinars, and general awareness programs. Through social media, television, and radio, the Ministry of Health and the IEDCR provide regular updates on the number of samples tested, infected patients, the most infected districts, the number of individuals quarantined in their homes, recovered patients, the death toll, and the availability of personal protective equipment (PPE). In addition, the COVID‐19 health bulletin is made public and distributed consistently.^52^ Community's increased awareness of the importance of receiving vaccinations due to these measures, an extremely high proportion of the population went vaccinated.

The global ties that Bangladesh has formed are beneficial. Not only vaccines but also medical equipment, PPE, and even oxygen were donated by friendly countries that border Bangladesh. These international alliances supported developing nations by immunizing them against COVID‐19 and other preventative measures.^54^ The Coalition for Epidemic Preparedness Innovations (CEPI), Global Alliance for Vaccine and Immunization (GAVI), WHO, and United Nations Children's Fund UNICEF collaborated on developing the COVID‐19 vaccine for Global Access (COVAX). It is essential to speed up the manufacturing and research of vaccines and ensure fair access and equal distribution for all countries.^46^ The greatest number of COVAX COVID‐19 vaccine doses were distributed to Bangladesh. COVAX was shipped to Bangladesh by UNICEF on June 1, 2021.^55^ Following that, UNICEF provided a donation of 190 million COVID‐19 immunizations and 26 ultra‐low temperature freezers to Bangladesh via COVAX.^43^, ^44^ By March 2023, a total of 355 million doses have been administrated, and over 151 million people got at least one dose of vaccine which is 91.77% of the total population.^7^, ^56^

## 6 CONCLUSION

During the COVID‐19 pandemic, the coronavirus had a detrimental effect on day‐to‐day living, particularly in the fields of health, finances, and the economy of Bangladesh. However, these long‐term effects of the coronavirus were decreased through mass vaccination. They have declined gradually due to Bangladesh's sustained commitment to maintaining excellent diplomatic connections with worldwide alliances, which helped attain the goal of vaccination level. In addition, the immunization program in Bangladesh during COVID‐19 was a resounding success since EPI was already well‐established throughout the nation. When all is considered, Bangladesh, despite its status as an emerging nation, has shown itself to be an outstanding leader in the field of global immunization.

## AUTHOR CONTRIBUTIONS

**Nazmunnahar**: Conceptualization; writing—original draft. **Bulbul Ahamed**: Conceptualization. **Md. Anamul Haque**: Conceptualization. **Md. Tanbir**: Conceptualization. **A. S. M. Roknuzzaman**: Writing—original draft; writing—review and editing. **Rapty Sarker**: Writing—original draft; writing—review and editing. **Md. Rabiul Islam**: Writing—review and editing.

## CONFLICT OF INTEREST STATEMENT

The authors declare no conflict of interest.

## TRANSPARENCY STATEMENT

The lead author Md. Rabiul Islam affirms that this manuscript is an honest, accurate, and transparent account of the study being reported; that no important aspects of the study have been omitted; and that any discrepancies from the study as planned (and, if relevant, registered) have been explained.

## Data Availability

Data sharing is not applicable to this article as no new data were created or analyzed in this study.
